# Supplementary material for: Strength can be controlled by edge dislocations in refractory high-entropy alloys
Source: Nat Commun. 2021 Sep 16;12:5474. doi: 10.1038/s41467-021-25807-w (PMC8446014; doi:10.1038/s41467-021-25807-w)
Supplement: Supplementary file 1 — Supplementary Information [file 41467_2021_25807_MOESM1_ESM.pdf]

Supplementary information for the manuscript

Strength Can be Controlled by Edge Dislocations in Refractory High-Entropy Alloys

1. Chanho Lee[1,2,§], Francesco Maresca[3,4*,§], Rui Feng[1,5,§], Yi Chou[6], T. Ungar[7], Michael Widom[8], Ke An[5], Jonathan D. Poplawsky[9], Yi-Chia Chou[6], Peter K. Liaw[1*],

2. and W. A. Curtin[4]

3. Department of Materials Science and Engineering, The University of Tennessee, Knoxville, TN 37996-2100, USA
4. Materials Science and Technology Division, Los Alamos National Laboratory, Los Alamos, NM 87545, USA
5. Engineering and Technology Institute (ENTEG), Faculty of Science and Engineering, University of Groningen, Groningen, 9474AG, Netherlands
6. Laboratory for Multiscale Mechanics Modeling, École Polytechnique Fédérale de Lausanne, CH-1015 Lausanne, Switzerland
7. Neutron Scattering Division, Oak Ridge National Laboratory, Oak Ridge, TN, 37831, USA
8. Department of Electrophysics, National Yang Ming Chiao Tung University, Hsinchu, 30010, Taiwan
9. Department of Materials Physics, Eötvös University, Budapest, P.O. Box 32, H-1518, Hungary
10. Department of Physics, Carnegie Mellon University, Pittsburgh, PA 15213, USA
11. Center for Nanophase Materials Sciences, Oak Ridge National Laboratory, Oak Ridge, TN, 37831, USA

*Corresponding author: f.maresca@rug.nl and pliaw@utk.edu

Table of Contents

Supplementary Figure 1.	SEM-BSE image of the homogenization-treated NbTaTiV refractory HEA
Supplementary Figure 2.	Mechanical properties of the homogenized NbTaTiV HEA obtained at 293 K, 973 K, and 1,173 K, respectively
Supplementary Figure 3.	Mechanical properties of the as-cast CrMoNbV HEA obtained at 293 K, 973 K, and 1,173 K, respectively
Supplementary Figure 4.	The analysis on dislocation types in a 15%-deformed NbTaTiV
Supplementary Figure 5.	Reciprocal diffraction elastic constants, $1/E_{\{hkl\}}$ and $v_{hkl}/E_{\{hkl\}}$, as a function of A_{hkl}, and fitting with the Kröner model
Supplementary Figure 6.	The dislocation core structures and diffraction patterns of strained NbTaTiV and CrMoNbV
Supplementary Figure 7.	TEM experiments showing dominance of edge dislocations in NbTaTiV and CrMoNbV at 1,173 K
Supplementary Figure 8.	Theory predictions for T = 1,300 K strength vs. composition and strength/density vs. composition
Supplementary Note 1.	Microstructure and chemical composition of the homogenized NbTaTiV HEA
Supplementary Note 2.	*In-situ* neutron diffraction and mechanical testing
Supplementary Note 3.	TEM Analysis of Burgers vector and character in NbTaTiV
Supplementary Table 1.	Normalized **g·b** values for possible Burgers vectors under the imaging conditions used in Supplementary Figure 4
Supplementary Note 4.	Analysis of elastic response
Supplementary Table 2.	The single-crystal macroscopic elastic constants, C_{11}, C_{12}, and C_{44}, the macroscopic Young's (E_M), shear (G_M), bulk (K_M) moduli, and Poisson's ratio (v) at 293 K, 973 K, and 1,173 K, respectively.
Supplementary Note 5.	Dislocation core structures of NbTaTiV and CrMoNbV
Supplementary Note 6.	TEM analysis of dislocation types in high-temperature-deformed NbTaTiV and CrMoNbV
Supplementary Note 7.	Finding high-temperature strengths in the whole Cr-Mo-Nb-Ta-V-W-Ti-Zr-Hf-Al composition space
Supplementary Table 3.	Single-element properties used for theoretical predictions
Supplementary Note 8.	Energetic competition within the Cr-Mo-W-Zr alloy system
Supplementary references	

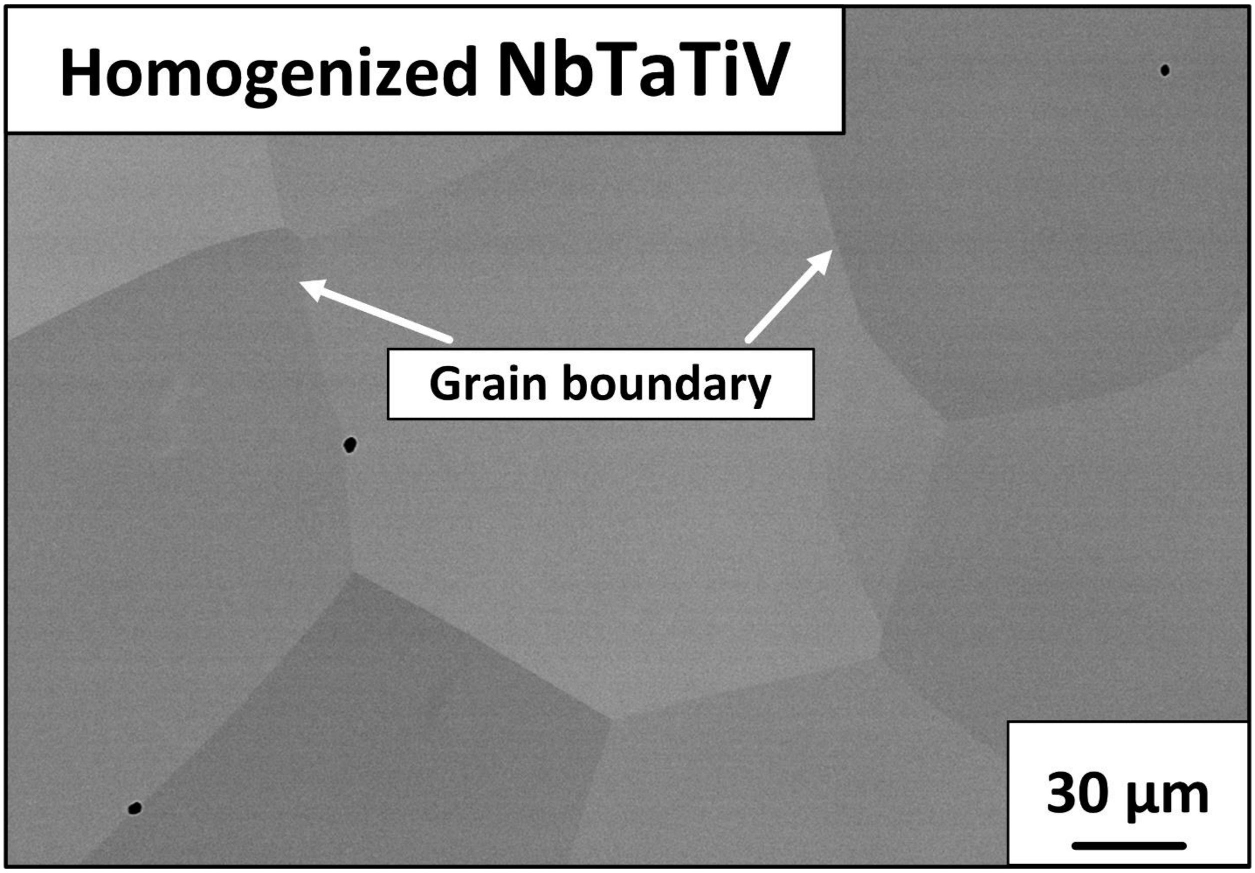

Supplementary Figure 1. SEM-BSE image of the homogenization-treated NbTaTiV refractory HEA.

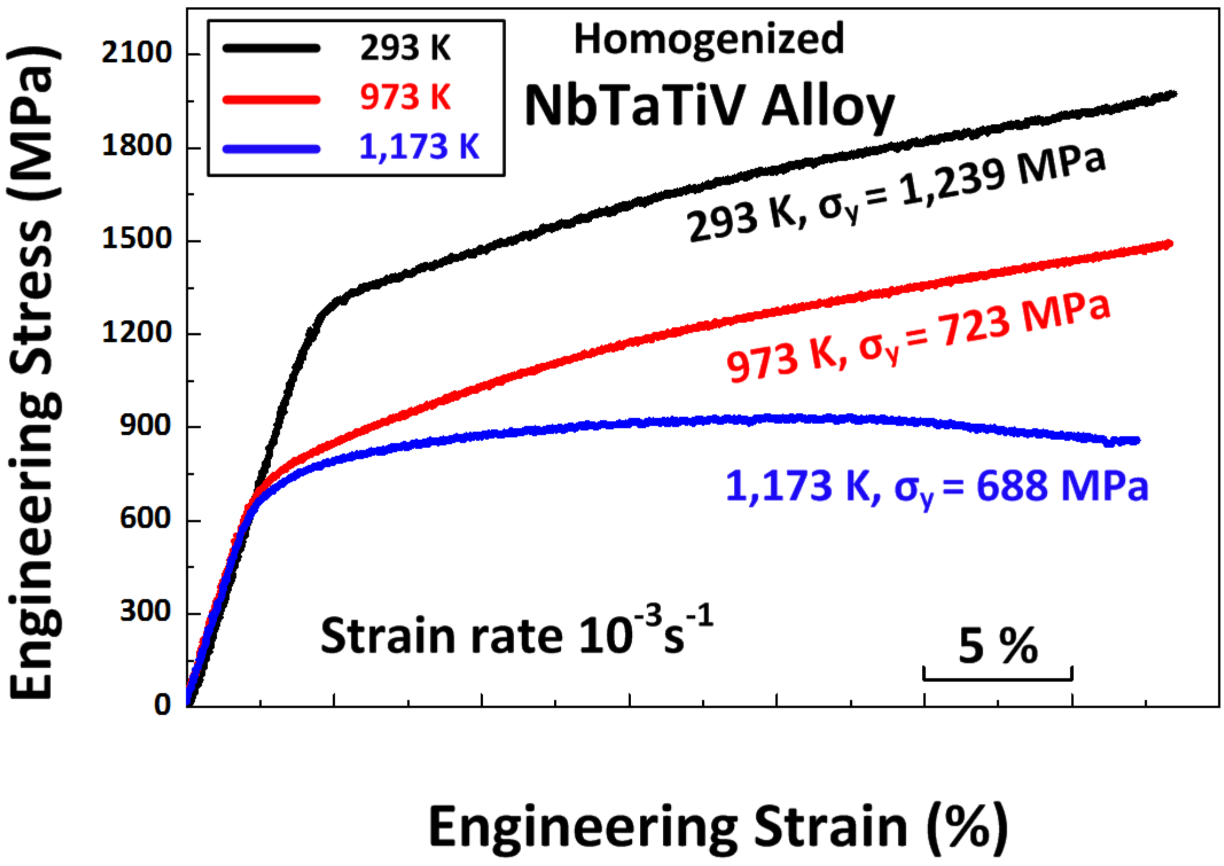

Supplementary Figure 2. Mechanical properties of the homogenized NbTaTiV HEA obtained at 293 K, 973 K, and 1,173 K, respectively.

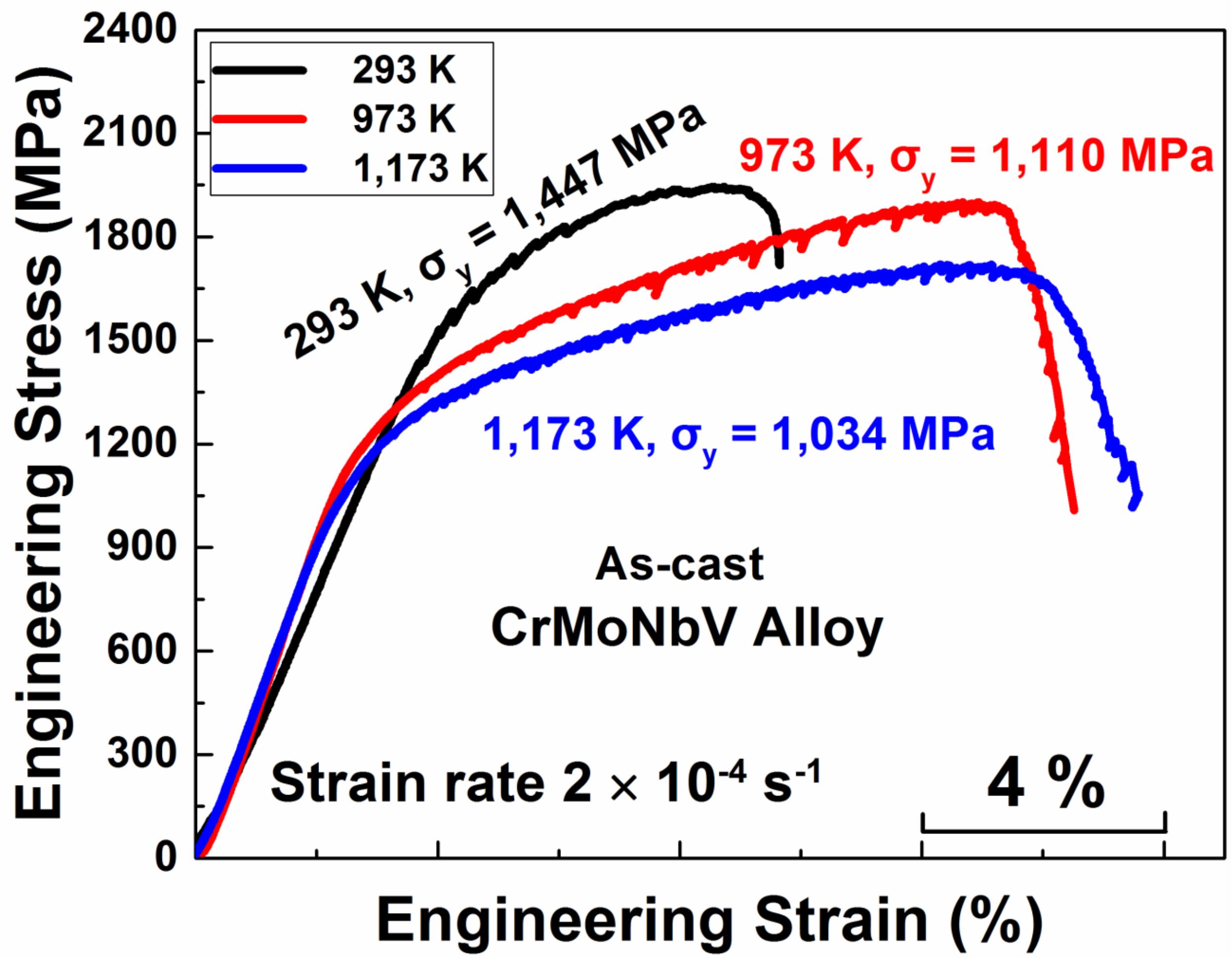

Supplementary Figure 3. Mechanical properties of the as-cast CrMoNbV HEA obtained at 293 K, 973 K, and 1,173 K, respectively.

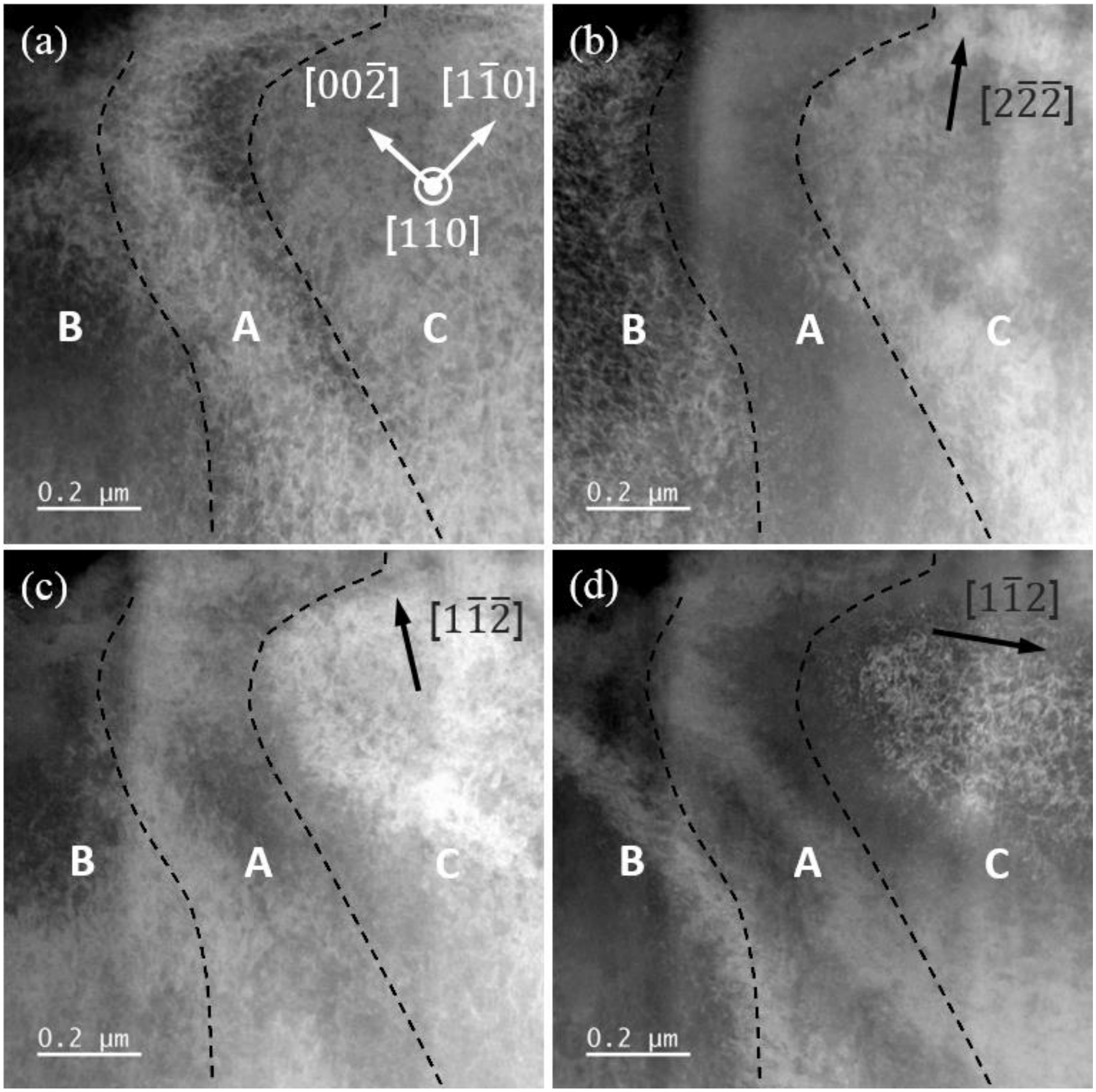

Supplementary Figure 4. The analysis on dislocation types in a 15%-deformed NbTaTiV. All the images were taken in the same region but different imaging conditions. The line marks separating the regions showed different contrasts in each imaging condition. **a**, Annular Dark-field (ADF) image viewing along the [110] directions. The two lowest indexed directions were marked with arrows. The ADF images viewing along the [110] and with **b**, [$2\bar{2}\bar{2}$], **c**, [$1\bar{1}\bar{2}$], and **d**, [$1\bar{1}2$] g vectors. The g vectors were marked on the images with arrows.

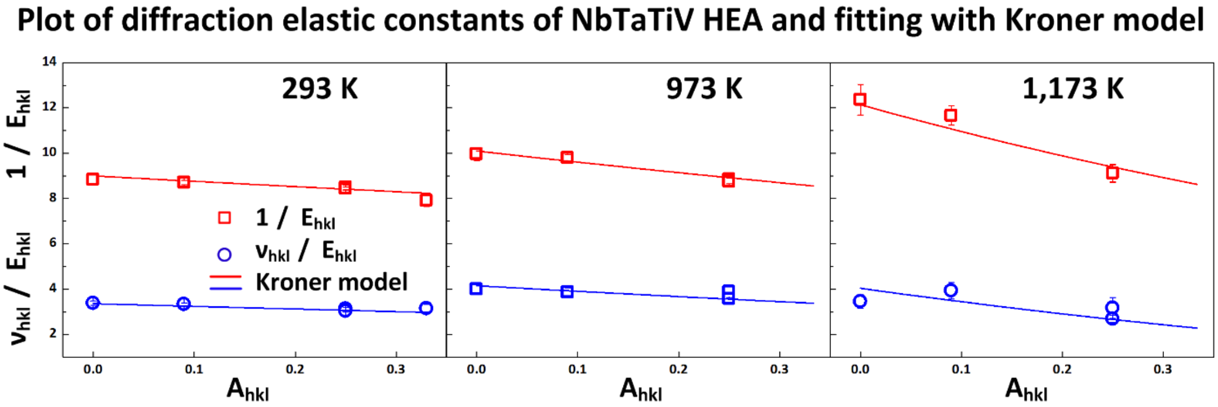

Supplementary Figure 5. Reciprocal diffraction elastic constants, $1/E_{\{hkl\}}$ and $\upsilon_{hkl}/E_{\{hkl\}}$, as a function of A_{hkl}, and fitting with the Kröner model.

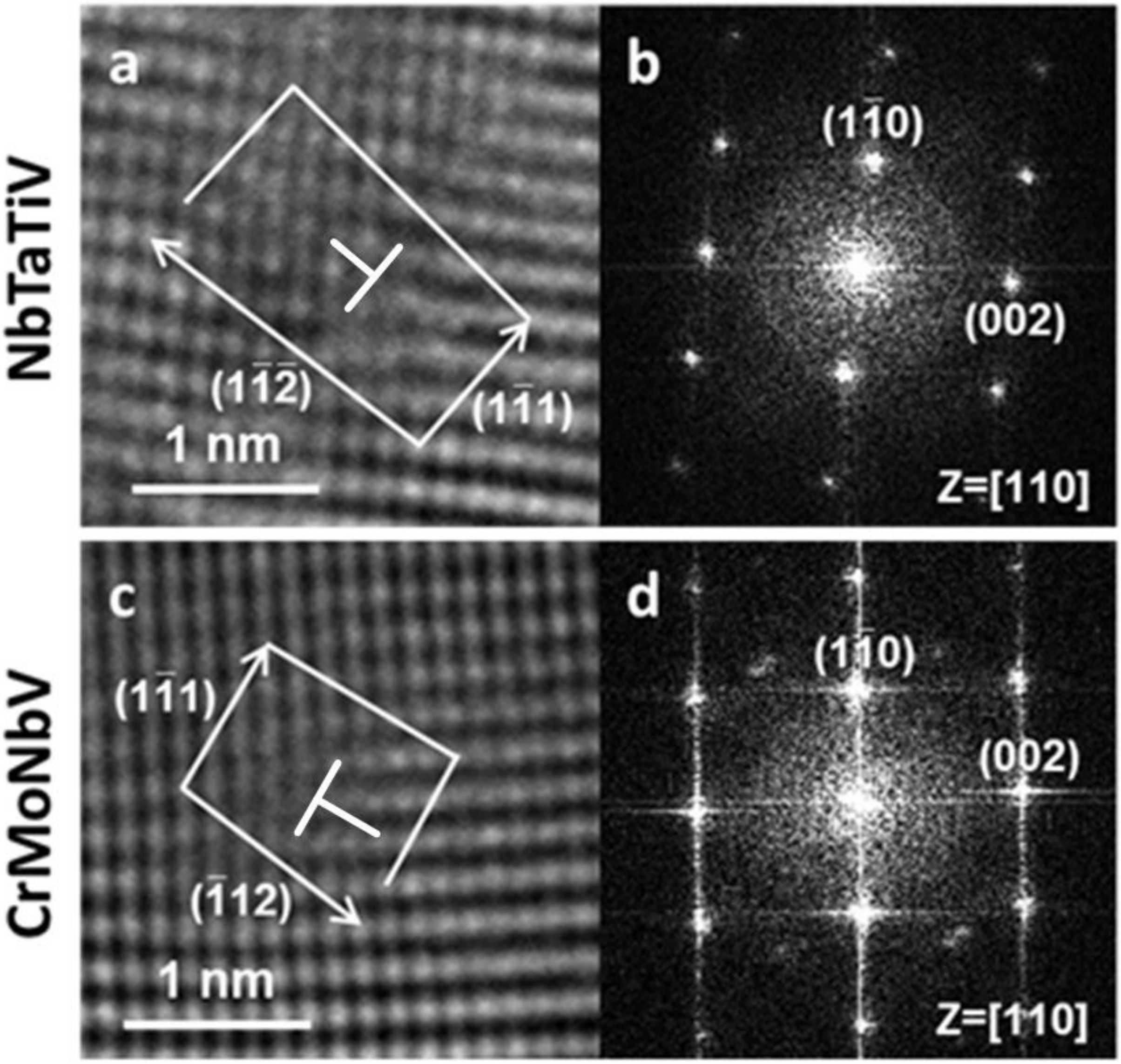

Supplementary Figure 6. The dislocation core structures and diffraction patterns of the strained NbTaTiV and CrMoNbV. **a**, **c**, the filtered high-resolution HAADF image of the 11.8 % - strained NbTaTiV and 4.2 % - strained CrMoNbV projected along the [110] direction as shown in the corresponded Fast Fourier transform (FFT) spectrum **b**, **d**, and the dislocation core was marked as "T". Based on the FFT spectrum, the extra ($1\bar{1}1$) plane can be directly observed, and the corresponding slip plane was on ($1\bar{1}\bar{2}$). The scale bar represented 1 nm.

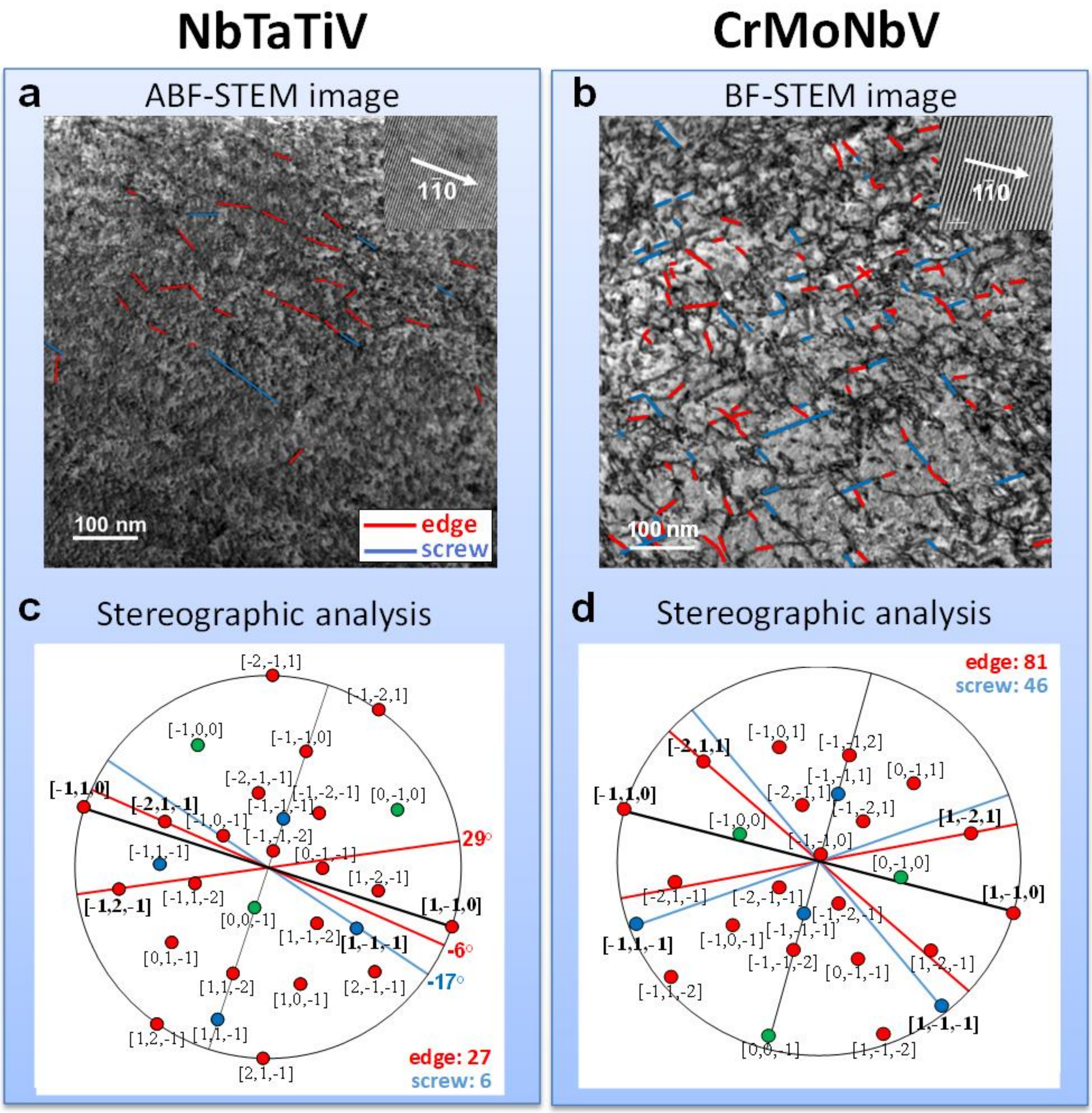

Supplementary Figure 7. TEM experiments showing the dominance of edge dislocations in NbTaTiV and CrMoNbV at 1,173 K. a, b, Bright-field (BF) STEM image of the 11.8 % - deformed NbTaTiV and 4.2 % - strained CrMoNbV at 1,173 K with a two-beam condition near the $Z = [113]$ or $Z = [110]$ and the $\vec{g} = (1\bar{1}0)$. Inset: HAADF image showing the $\vec{g} = (\bar{1}10)$ plane. Dislocation lines are indicated by red (edge) or blue (screw) lines corresponding to different angles with respect to the $[1\bar{1}0]$ direction. Due to the high-temperature deformation, many

dislocation loops can be observed, which may obscure some dislocation lines. **c, d**, Stereographic projection of possible dislocation-line directions, where $[1\bar{1}0]$ has been aligned with **a** and **b**. The degrees indicate the angle with respect to the $[1\bar{1}0]$ direction. In NbTaTiV, 27 edge and at most 6 screw dislocations are identified. In CrMoNbV, 81 edge and at most 46 screw dislocations are identified.

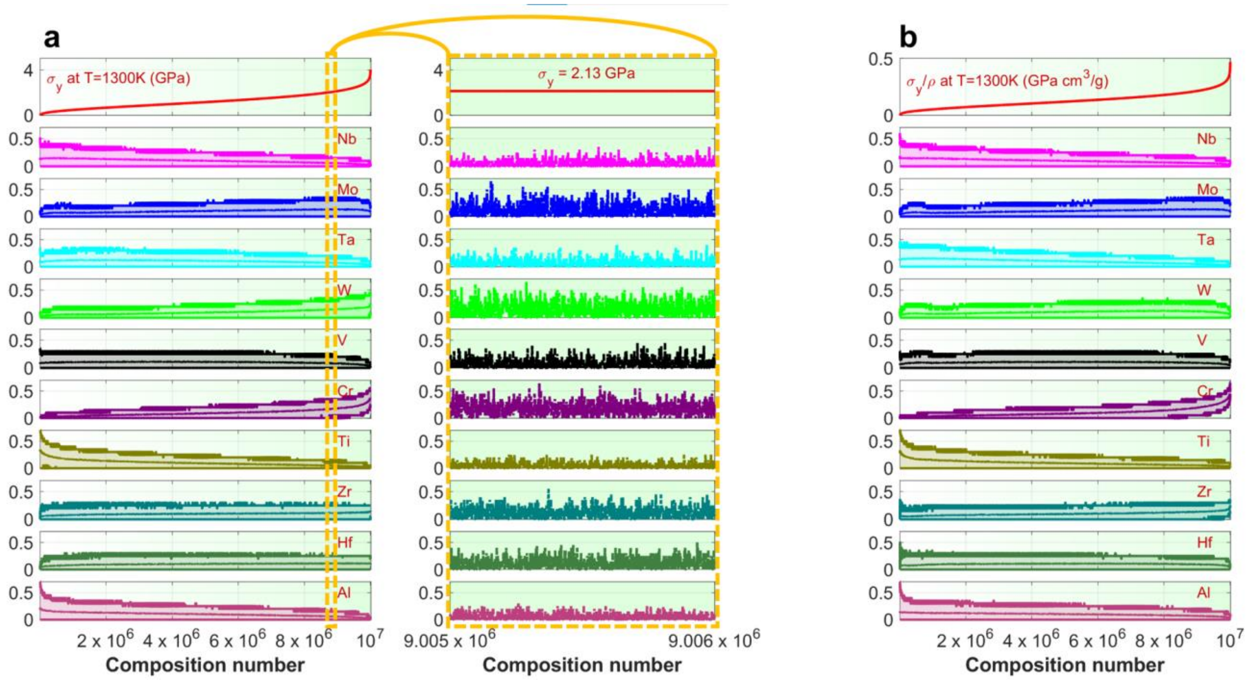

Supplementary Figure 8. a, Theory predictions for T = 1,300 K strength vs. composition. As detailed in the Supplementary Note 7, computations of yield strengths have been performed for > 10,000,000 alloys. The alloys have been assigned with an increasing number as a function of the increasing strength. Thus, the lowest strength alloy is the number 1, and the highest strength alloy is the number 10,003,049. For better visualization, we have grouped the alloys into bins containing 1,000 compositions each. Here, as zoom-in is shown over one bin, which includes the compositions between 9,005,000 and 9,006,000. In the left panel, the shaded areas indicate the compositions between the 10th and 90th percentiles, and the middle line indicates the average of the composition (by considering 1,000 compositions per bin). Thus, the non-dimensional y axis is the elemental concentration. The zoom-in shows the raw data, which are characterized by large fluctuations in the composition for approximately the same strength. In the example presented, there are 1,000 compositions that can attain ~ 2.13 GPa at T = 1,300 K, and all elements of the Nb-Mo-Ta-W-V-Cr-Ti-Zr-Hf-Al compositional space can be used. The elements that are typically in the largest concentrations are Cr, W, and Mo, followed by Zr and Hf. **b**, Theory predictions for

T = 1,300 K strength/density vs. composition. The plot is constructed in the same way as the panel **a**, by ranking the compositions from the lowest to the largest strength/density ratio.

Supplementary Note 1. Microstructure and chemical composition of the homogenized NbTaTiV HEA

Supplementary Figure 1 shows the scanning-electron microscopy (SEM) back-scattered electrons (BSE) images of the homogenized NbTaTiV HEA. The simple solid-solution microstructure was clearly observed with no formation of second phases or elemental segregation within grains and grain boundaries. The chemical composition of the homogenization-treated sample was identified by the APT analysis, which is close to the nominal composition ($Nb_{24.778}Ta_{23.471}Ti_{24.893}V_{24.929}C_{0.072}Ga_{0.278}Al_{0.002}N_{0.424}O_{1.153}$ on the average of the atom fractions). The CrMoNbV alloy shows a typical dendritic and interdendritic microstructure with a body-centered-cubic (BCC) structure, resulting from the different melting points of constituent elements. The detailed microstructure information of the CrMoNbV alloy can be found in the previous work[1].

Supplementary Note 2. *In-situ* neutron diffraction and mechanical testing

The lattice strains (ε_{hkl}) were calculated from the variation of the diffraction-peak positions, during loading by the following equation:

$$\varepsilon_{hkl} = \frac{d_{hkl} - d_{hkl}^{0}}{d_{hkl}^{0}} \tag{1}$$

where $d_{hkl}$is the *hkl* lattice spacing as a function of the applied stress, and d_{hkl}^{0} is the reference *hkl* lattice spacing under the unloaded state. The lattice-strain evolution can provide the $\{hkl\}$ plane-specific lattice strain during deformation. The evolution of lattice strains in the elastic region exhibits that the change of lattice strains is not dependent on the grain orientation, i.e., all oriented-grains [$\{110\}, \{200\}, \{211\},$ and $\{310\}$] present almost the similar elastic-lattice strains with an identical level of the applied stress and maintain the linear response, indicating the elastic isotropy.

Supplementary Figure 2 exhibits the compressive engineering stress-strain curves for the homogenized NbTaTiV HEA at elevated temperatures. This alloy indicates the high yield strength (σ_y) of 1,239 MPa without the occurrence of fracture until the compressive strain of 30 % at 293 K. As the temperature is increased to 1,173 K, the yield strength gradually reduces to 688 MPa with the maintenance of the excellent compressive plasticity. However, work softening is clearly observed when the sample is plastically deformed at 1,173 K. Supplementary Figure 3 displays the compressive engineering stress-strain curves for the as-cast CrMoNbV HEA at 293 K, 973 K, and 1,173 K. One can notice that this alloy exhibits a very high yield strength at room temperature (1,447 MPa). The yield strength of this alloy retains a high value (> 1,000 MPa) up to 1,173 K. The detailed values of yield strengths for the homogenized NbTaTiV and as-cast CrMoNbV alloys at 293 K, 973 K, and 1,173 K are noted in Supplementary Figures 2 and 3.

Supplementary Note 3. TEM Analysis of Burgers vector and character in NbTaTiV

The TEM contrast of a dislocation is related to **g·b,** and when |**g**·**b**|/gb > 1/3, the contrast is visible in the TEM. Supplementary Table 1 summarizes relevant possible combinations of Burgers vectors and their contrasts with respect to the **g** vectors that were $[2\bar{2}\bar{2}]$, $[1\bar{1}\bar{2}]$, and $[1\bar{1}2]$, which were selected for our investigation. For all <100> Burgers vectors, |**g**·**b**|/gb > 1/3, and such dislocations would show contrasts in all the images. For some <110> and <111> Burgers vectors, |**g**·**b**|/gb < 1/3 for some **g**, highlighted as red in the Table. Furthermore, both Burgers vectors, $[1\bar{1}1]$ and $[101]$, have the same contrast conditions (highlighted by gray shading in the Table) and cannot be distinguished. Furthermore, both Burgers vectors, $[1\bar{1}\bar{1}]$ and $[10\bar{1}]$, have the same contrast conditions (highlighted by blue in Supplementary Table 1) and so also cannot be distinguished. Only the $[111]$ Burgers vector presented dislocation contrasts for **g** of $[1\bar{1}\bar{2}]$ and $[1\bar{1}2]$, and invisibility for $[2\bar{2}\bar{2}]$. The [110] Burgers vector is the only case that shows no dislocation contrasts for all **g** vectors. Based on these various conditions, we can interpret our STEM data showing dislocation contrasts for the various imaging conditions (**g** vectors). Not all dislocations can be uniquely determined but our subsequent stereographic projections based on <111> Burgers vectors provides complementary data that the dislocations have the <111> Burgers vectors.

Supplementary Figure 4 shows the STEM-ADF images viewed along the [110] direction for different conditions. Regions A, B, and C are indicated only for references in the discussion. In Supplementary Figure 4a, all diffraction spots are included, and hence, all dislocations are visible. Supplementary Figure 4b showed the STEM ADF image for the $[2\bar{2}\bar{2}]$ g vector, and many of the dislocations in Region A are now invisible. In Region B, nearly horizontal dislocation lines appear. In Region C, the contrast is blurred but the top-half area is grayer and shows less dislocation contrast. Supplementary Figure 4c has the $[1\bar{1}\bar{2}]$ g vector, and Region A presents two

different contrasts, with the top area and bottom-left corner exhibiting lighter contrast, and the center area demonstrating darker contrast. The bottom-half area in Region B and the top-half area in Region C also show contrast. Supplementary Figure 4d has the $[1\bar{1}2]$ g vector, and Region A presents lighter contrast in the top half and the bottom-left corner while Region C exhibits lighter contrast in the top half with obvious vertical dislocation lines.

According to Supplementary Table 1, the contrast conditions observed for the top and bottom-left-corner in Region A and for the top half in Region C are only in agreement with [111] Burgers vector (contrasts for $[1\bar{1}\bar{2}]$ and $[1\bar{1}2]$, no contrast for $[2\bar{2}\bar{2}]$). Region B provides the information on the dislocation character, as discussed below. The center area in Region A matches with either $[1\bar{1}\bar{1}]$ or $[10\bar{1}]$ Burgers vectors (contrast for $[1\bar{1}2]$, no contrasts for $[2\bar{2}\bar{2}]$ or $[1\bar{1}\bar{2}]$). Therefore, only the <111> type Burgers vectors are consistent with the contrasts in all regions for all g vectors. While $[10\bar{1}]$ is theoretically possible in Region A, it is absent in other regions and would be an unusual Burgers vector for slip in a BCC crystal. We conclude that the Burgers vectors in the NbTaTiV alloy are of the <111> type that is the well-established Burgers vector in elemental and dilute BCC alloys.

The TEM or STEM images can provide some further indication regarding the dislocation character, as described below. Recall that the line directions for edge and screw dislocations are perpendicular and parallel to **b**, respectively. Therefore, if the **g** vector is parallel to the dislocation line, screw dislocations will have the largest contrast, and edge dislocations will not be visible. If the projection of the dislocation line onto the [110] plane (axis of viewing) is aligned with **g**, then the contrast can determine the character. In addition, if the **g** vector is perpendicular to the dislocation line, screw dislocations will not show any dislocation contrast, but edge dislocations may present dislocation contrast when $|\mathbf{g}\cdot\mathbf{b}|/gb < 1/3$.

For example, a $[1\bar{1}\bar{1}]$ screw dislocation has a $[1\bar{1}\bar{1}]$ line direction, while an [111] edge dislocation has a $[2\bar{1}\bar{1}]$ line direction. The projections of these two dislocations onto the (110) plane lead to projected line directions that differ by only 10.02 degrees while the angle between the Burgers vectors is 70.52 degrees. Looking closely at Region A of Supplementary Figure 4a, we can observe a predominance of near-vertical dislocation lines. If these near-vertical dislocation lines are a screw type, the Burgers vector should be $[1\bar{1}\bar{1}]$ and on the (110) plane. For g = $[2\bar{2}\bar{2}]$, which is near vertical, and such a $[1\bar{1}\bar{1}]$ screw dislocation should be clearly visible. If the line is invisible, then the dislocation must be of edge character, which is the situation seen in Region A for Supplementary Figure 4b.

As another example, near-$[1\bar{1}2]$ dislocation lines in Region B of Supplementary Figure 4b with **g** = $[2\bar{2}\bar{2}]$ are observed. A screw dislocation should show no contrast but here the dislocation lines are clearly visible. Similarly, near $[1\bar{1}\bar{2}]$ and $[2\bar{1}\bar{1}]$ [the projected vector is $[3\bar{3}\bar{2}]$ on the (110) plane], dislocation lines are observed in Supplementary Fig. 4d with g = $[1\bar{1}2]$ and match with the edge character.

The **g·b** contrast cannot reveal the character of all the observed dislocations. The stereographic projection method is thus used for the better determination of the overall distribution of the dislocation character, as shown in the main text. The ratio of dislocation types was not weighted to the dislocation-line length because in the TEM measurement, the observed dislocation lines were from the projection of dislocation lines, and the length on the image was not the actual dislocation length. Therefore, the length-weighted dislocation type ratio may not represent the actual dislocation-type ratio. Rather, the number ratio should be closer to the actual dislocation-type ratio.

b		$g_{[2\bar{2}\bar{2}]}\cdot b$	$g_{[1\bar{1}\bar{2}]}\cdot b$	$g_{[1\bar{1}2]}\cdot b$
<111>	[111]	-0.33	-0.47	0.47
	$[1\bar{1}1]$	0.33	0.00	0.94
	$[1\bar{1}\bar{1}]$	1.00	0.94	0.00
<110>	[110]	0.00	0.00	0.00
	[101]	0.00	-0.29	0.87
	$[10\bar{1}]$	0.82	0.87	-0.29
	$[1\bar{1}0]$	0.82	0.58	0.58
<100>	[100]	0.58	0.41	0.41
	[010]	-0.58	-0.41	-0.41
	[001]	-0.58	-0.82	0.82

Supplementary Table 1. Normalized **g·b** values for possible Burgers vectors under the imaging conditions used in Supplementary Figure 4. Absolute values of |**g·b**|/gb < 1/3 are invisible and are highlighted by red. Pairs of Burgers vectors with the |**g·b**|/gb shaded in either gray or blue are not distinguishable under these conditions.

Supplementary Note 4. Analysis of elastic response

Supplementary Figure 5 shows the reciprocal diffraction elastic constants ($1/E_{hkl}$ and v_{hkl}/E_{hkl}), calculated by the Kröner model[2], plotted as a function of the elastic-anisotropy factor, $A_{hkl} = \left\{\frac{h^2k^2+k^2l^2+l^2h^2}{(h^2+k^2+l^2)^2}\right\}$, at both room and elevated temperatures[2-4]. It is found that the theoretical Kröner model fits the experimental data very well at all temperatures. Based on this good agreement with the *in-situ* neutron experimental data, the single-crystal elastic constants (C_{ij}), macroscopic bulk modulus (K_M), and shear modulus (G_M) were calculated, using the Kröner's self-consistent model[3, 4]:

$$G_K^3 + \alpha G_K^2 + \beta G_K + \gamma = 0 \quad (2)$$

where G_K is the diffraction shear modulus, and α, β, and γ are constants given by [3]:

$$\alpha = \frac{3\{3K_M+4[\mu+3(\eta-\mu)A_{hkl}]\}}{8} - \frac{(2\eta+3\mu)}{5} \quad (3)$$

$$\beta = \frac{3K_M[\mu+3(\eta-\mu)A_{hkl}]}{4} - \frac{3(6K_M\eta+9K_M\mu+20\eta\mu)}{40} \quad (4)$$

$$\gamma = -\frac{3K_M\eta\mu}{4} \quad (5)$$

where K_M is the bulk modulus, which is given by $(C_{11} + C_{12})/3$, η is $(C_{11} - C_{12})/2$, and μ is equal to C_{44}. The value of the isotropic macroscopic shear modulus, G_M, becomes the diffraction shear modulus, G_K, in Eq. (2), by substituting 0.2 for A_{hkl}, if averaging over all orientations. Then, the isotropic macroscopic Young's modulus, E_M, can be calculated by the equation:

$$E_M = \frac{9G_MK_M}{G_M+3K_M} \quad (6)$$

The calculated single-crystal elastic constants (C_{ij}), macroscopic Young's (E_M), shear (G_M), bulk (K_M) moduli, and Poisson's ratio (v) at room and elevated temperatures are listed in Supplementary Table 2.

Parameters / Conditions	C_{11} (GPa)	C_{12} (GPa)	C_{44} (GPa)	C'	E_M (GPa)	G_M (GPa)	K_M (GPa)	v
**293K**	196.79	121.44	46.74	37.68	117.3	42.9	146.6	0.368
**943K**	184.33	122.52	45.93	30.91	107.9	39.3	143.1	0.376
**1,173 K**	134.87	76.78	46.65	29.05	102.3	38.7	96.1	0.325

Supplementary Table 2. The single-crystal macroscopic elastic constants, C_{11}, C_{12}, and C_{44}, the macroscopic Young's (E_M), shear (G_M), bulk (K_M) moduli, and Poisson's ratio (v) at 293 K, 973 K, and 1,173 K, respectively.

Supplementary Note 5. Dislocation core structure of NbTaTiV and CrMoNbV

Supplementary Figures 6a and 6c shows the atomic-resolution HAADF images of the 11.8 % - strained NbTaTiV and 4.2 % - strained CrMoNbV. Both samples presented the extra $(1\bar{1}1)$ plane as indicated in Supplementary Figures 6a and 6c with "T", and the dislocation line was along the viewing direction [110]. It implied that the dislocations had a $(1\bar{1}1)$ Burgers vector and $(1\bar{1}\bar{2})$ slip plane. By the definition of the dislocation-core width, which was the distance between two atomic columns site with ±1/4 b Burgers vectors displacements on the extra plane, the corresponding values were 1.2 nm and 1.6 nm for NbTaTiV and CrMoNbV, respectively.

Supplementary Note 6. TEM analysis of dislocation types in high-T deformed NbTaTiV and CrMoNbV

Supplementary Figures 7a and 7b presents the BF-STEM images of 1,173 K - deformed NbTaTiV and CrMoNbV viewed along the [113]. The background was not as clear as in the RT case in Figures 2e and 3e because the high-temperature deformation facilitated the nucleation of a large number of dislocation loops, which may blur some of the dislocation lines. Under the careful examination, the dislocation lines were marked, and the types were determined by aligning the stereographic projection according to the zone axis and the inset HAADF images in Figures 2e and 3e.

For NbTaTiV, the zone axis was [113] and $\vec{g} = (1\bar{1}0)$. The red dislocation lines were edge dislocations, and the blue dislocation lines could be either screw dislocations with a <111> dislocation line vector or edge dislocations with a <110> dislocation line vector. Here, we categorized all blue dislocation line to be screw dislocation, and there were 27 edge and 6 screw dislocations. The ratio of edge dislocations was 82 %. The actual ratio of edge dislocation could be even higher.

For CrMoNbV, the same method was applied, and the zone axis was [110] and $\vec{g} = (1\bar{1}0)$. Under this condition, the dislocation type can be easily distinguished. There were 81 edge dislocations and 46 screw dislocations. The ratio of edge dislocations was 64 %.

The ratio of edge dislocation increased in both NbTaTiV and CrMoNbV at a high temperature, which can further support our claim that edge dislocations played the key role in high temperature strengthening.

Supplementary Note 7. Finding high-temperature strengths in the whole Cr-Mo-Nb-Ta-V-W-Ti-Zr-Hf-Al composition space.

Figures 4b in the main text, as well as Supplementary Figure 7, presents the predictions of the reduced theory (see the main text) as a function of the composition for > 10,000,000 compositions in the whole Cr-Mo-Nb-Ta-V-W-Ti-Zr-Hf-Al space. The analytical theory (Eqs. 2 and 3 with Eqs. 4 and 5) has been used to compute yield strengths of over 10 million compositions in the whole Nb-Mo-Ta-W-V-Cr-Ti-Zr-Hf-Al family, at T = 1,300 K. Thus, the outcome of the calculations is a large table with > 10 M entries (to be precise, 10,003,049 compositions). Each entry records the composition and the yield strength (Figs. 4b and Supplementary Figure 8a) or the ratio between the yield strength and density (Supplementary Figure 8b). A label has been assigned to the compositions, from the lowest to the highest strength alloys. Thus, the number 1 is the composition with the lowest strength, and the highest strength is the number 10,003,049. By plotting the yield strength *vs.* composition, the top curve in Supplementary Fig. 8a is obtained. The curve is naturally "smooth" because the alloys have been numbered from the lowest to the highest strength (or strength/weight, Supplementary Figure 8b).

By following the alloy ranking from the lowest to the highest strength (or strength/ratio in Supplementary Figure 8b), it is known that on top of the yield strength, what is the alloy composition. By plotting directly, the Nb, Mo, etc. contents as a function of the alloy label (the weakest to strongest), the composition *vs.* alloy number is not a smooth function, but is rather noisy, as presented in the zoom-in of Supplementary Figure 8a. This is because one can find alloys that have similar yield strengths, but very different compositions. Therefore, for the sake of the visualization, the elemental content vs. compositions plot has been "smoothened", by following this operation: take the first 1,000 lowest strength compositions. Compute the average Nb, Mo, ...

concentrations among these first 1,000 compositions, and store the result. Take the next 1,000 compositions, compute the average, and store the result. Proceed until one covers all the alloy compositions. Thus, as a final result, one obtains the average contents of Nb, Mo, etc... per “bins” of 1,000 compositions. Hence, the bin-averaged composition can be plotted as a function of the bin number, which goes from 1 to > 10,000. During this averaging operation, the “noise” is lost. In order to assess how much an element can be varied within a bin, to obtain a similar yield strength, the 10th and 90th percentiles of the concentrations, per bin, have been computed. The plot reports the shaded area between 10th and 90th percentiles and, in Supplementary Figure 8, also the average, per bin, of the concentration of each element. Therefore, the y axis of the composition plots for Nb, Mo, Ta, etc. is dimensionless, because it corresponds to the elemental concentration. The screening is performed, using as the input of the single-elemental atomic volumes, elastic constants, and densities, all listed in Supplementary Table 3. The alloy values are then computed, employing the rule of mixtures of the elemental values (see the main text).

For the case of Al, the atomic volume is 14,075 $Å^3$, based on the work by Chen et al.[5]. The atomic volumes of all other elements are the same, as reported in the Ref.[6]. For the Ti and Zr, the values are obtained by extrapolating high-temperature (high-T) measurements to room temperature (RT), while Hf is obtained, employing the Vegard’s law on Hf-HEAs. The atomic volumes adopted for Ti, Zr, and Hf are similar to those estimated in Ref.[7], which were instead obtained by extrapolating the elemental values from binary alloys in the literature.

The cubic elasticity constants of the BCC Al are assumed to be equal to the FCC values. The Ti, Zr, and Hf values are taken from high-T phonon measurements (at 1,293 K, 1,188 K, and 2,073 K, respectively), see in Ref.[8-10].

The public repository with the raw data and a MATLAB code to predict the yield strengths of BCC HEAs as a function of temperature can be found from https://archive.materialscloud.org/record/2021.65 and doi: 10.24435/materialscloud:fs-27. The detailed description of the METLAB code and Materials Cloud are shown below. We have also included the MATLAB code as a separate Supplementary Information file.

Prediction of yield strengths in refractory body-centered-cubic High Entropy Alloys

How to cite this record

Francesco Maresca, Chanho Lee, Rui Feng, Yi Chou, Tamas Ungar, Michael Widom, Jonathan Poplawsky, Yi-Chia Chou, Peter Liaw, William Curtin, Prediction of yield strength in refractory body-centered-cubic High Entropy Alloys, Materials Cloud Archive 2021.65 (2021), doi: 10.24435/materialscloud:fs-27.

Description

Energy efficiency is motivating the search for new high-temperature metals. Some new body-centered-cubic random multicomponent "high-entropy alloys (HEAs)" based on refractory elements (Cr-Mo-Nb-Ta-V-W-Hf-Ti-Zr) possess exceptional strengths at high temperatures, but the physical origins of this outstanding behavior are not known.

Here, using a recent mechanistic theory, we have computed the high-temperature (T = 1,300 K) yield strengths based on solute strengthening of over 10 million alloys within the whole Al-Cr-Mo-Nb-Ta-V-W-Hf-Ti-Zr alloy family. In addition, the yield strength/density has been computed. This database enables the efficient search of new alloys with exceptional high-temperature strengths.

Materials Cloud sections using this data

No Explore or Discover sections associated with this archive record.

License

Files and data are licensed under the terms of the following license: Creative Commons Attribution 4.0 International.

Metadata, except for email addresses, are licensed under the Creative Commons Attribution Share-Alike 4.0 International license.

External references

Preprint (Preprint where the data is discussed)

F. Maresca, C. Lee, R. Feng, Y. Chou, T. Ungar, M. Widom, K. An, J. Poplawsky, Y.-C. Chou, P. Liaw., W. Curtin, arXiv:2008.11671 (2020)

Journal reference (Paper in which the theory is described)

F. Maresca, W. Curtin, Acta Mater. 182, 235-249 (2020) doi:10.1016/j.actamat.2019.10.015

Keywords

High-entropy alloys, Solute strengthening, High-temperature strength, ERC, EPFL

	Volume (Å^3)	C_{11} (GPa)	C_{12} (GPa)	C_{44} (GPa)
**Al**	14.075	105.6	63.9	28.53
**Cr**	12.321	339.8	58.6	99
**Hf**	22.528	131	103	45
**Mo**	15.524	450.02	172.92	125.03
**Nb**	17.952	252.7	133.2	30.97
**Ta**	17.985	266.32	158.16	87.36
**Ti**	17.387	134	110	36
**V**	14.020	232.4	119.36	45.95
**W**	15.807	532.55	204.95	163.13
**Zr**	23.02	104	93	38

Supplementary Table 3. Single-element properties used for theoretical predictions.

Supplementary Note 8. Energetic competition within the Cr-Mo-W-Zr alloy system

In order to achieve a BCC solid solution, we require the free energy to be lower than competing phases. Two factors enter consideration, enthalpy and entropy. We will compute enthalpies within the Cr-Mo-W-Zr quaternary alloy system utilizing density functional theory (DFT). In addition to the pure elements and random solid solutions, the competing phases are the ordered BCC phase of Pearson type, cP2 (B2), and Laves phases of Pearson types, hP12, hP24, and cF24 (Strukturbericht C14, C36, and C15), Mo_3Zr.cP8, and W_5Zr_3.tI32.

Our DFT calculations employ the plane-wave code, VASP[11], in the Perdew-Burke-Ernzerhof (PBE) generalized gradient approximation[12] with an energy cutoff of 300 eV and k-point densities sufficient to converge energies to within 1 meV/atom. We fully relax atomic positions and lattice parameters and consider antiferromagnetic spin polarization in the case of the elemental Cr. Enthalpies are calculated, relative to pure elements. Stable phases lie on the convex hull of enthalpies. Instability energies, ΔE, are defined as the height above the convex hull[13].

The BCC solid solutions are represented as randomly-occupied supercells containing N_1 – N_4 atoms of the four different species. A total of

$$\Omega(N_1, N_2, N_3, N_4) = \frac{(N_1+N_2+N_3+N_4)!}{N_1!N_2!N_3!N_4!} \quad (7)$$

Such configurations exist from which we take $N_{samples}$ = 20 representative configurations in order to obtain a distribution of instability energies, ΔE_k. Our energies, ΔE_k, range from 232 up to 314 meV/atom, with an average value of 266 and standard deviation of 15 meV/atom. The energies define a partition function[14]

$$Z = \frac{\Omega(N_1,N_2,N_3,N_4)}{N_{samples}} \sum_{k=1}^{N_{samples}} e^{-E_k/k_B T} \quad (8)$$

and its associated free energy, $F = -k_B T ln(Z)$, in which varying degrees of short-range chemical order are appropriately weighted. The BCC solid-solution gains stability, relative to competing phases above the temperature, T_0, at which F vanishes. Vibrational and electronic entropies are neglected, as these are found to be small effects, relative to the entropy of chemical substitution when comparing phases of similar structures (*i.e.*, BCC phase)[15].

For the equiatomic CrMoWZr, we predict phase separation at low temperatures into a mixture of BCC-Cr, Cr_2Zr.cF24, W_2Zr.cF24, and BCC-Mo. The transition to a single-phase BCC structure occurs at T_0 = 2,300 K. The melting temperature is not precisely known. We estimate it as 2,300 K by averaging the melting temperatures of the six equiatomic binaries. Thus, we predict the equiatomic BCC phase to be unstable at all temperatures below melting. Because Laves phases are major competitors to the HEA, we estimated the free energy of the cF24 Laves phase assuming a concentration, Mo_2Zr_6, on site 8a and $Cr_6Mo_4W_6$, on site 16d. This structure lies 82 meV/atom above the convex hull, suggesting that it could be stabilized by the entropy of mixing on the sublattices above T_0 = 1,300 K.

Cr and Zr are especially prone to the Laves-phase formation. Hence, we investigated the effect of moving off-stoichiometry on $CrMo_2W_2Zr$ within a 24-atom supercell. Because the composition moves away from the Cr_2Zr and W_2Zr Laves phases, the distribution of ΔE_k values shifts downward by approximately 100 meV/atom, and we predict the formation of a single-phase BCC structure at T_0 = 1,600 K. At the same time, the melting temperature should rise because the composition is enriched in elements, Mo and W, whose melting temperatures are high. Thus, we obtain a thermally-stable non-stoichiometric HEA over a wide temperature range. However, this analysis considers only a subset of potentially-competing phases that omit binary and ternary solid solutions. Hence, further investigation will be required to validate this prediction.

The situation is similar for CrHfMoW, whose phase separates at low temperatures into a mixture of BCC phases plus the Laves phase, HfW_2. The transition to a single BCC phase occurs at T_0 = 2,400 K, compared with our estimated melting temperature of 2,100 K. The high-entropy Laves phase is stabilized above T_0 = 1,350 K. At the composition, $CrHfMo_2W_2$, the predicted T_0 drops to 1,200 K, while the melting temperature rises.

Supplementary references

1. Feng R, Liaw PK, Gao MC, Widom MJnCM. First-principles prediction of high-entropy-alloy stability. **3**, 50 (2017).

2. Kröner E. Berechnung der elastischen Konstanten des Vielkristalls aus den Konstanten des Einkristalls. *Zeitschrift für Physik* **151**, 504-518 (1958).

3. De Wit R. Diffraction elastic constants of a cubic polycrystal. *Journal of applied crystallography* **30**, 510-511 (1997).

4. GnaÈupel-Herold T, Brand PC, Prask HJ. Calculation of single-crystal elastic constants for cubic crystal symmetry from powder diffraction data. *Journal of applied crystallography* **31**, 929-935 (1998).

5. Chen H, *et al.* Contribution of lattice distortion to solid solution strengthening in a series of refractory high entropy alloys. *Metallurgical Materials Transactions A* **49**, 772-781 (2018).

6. Yin B, Maresca F, Curtin W. Vanadium is an optimal element for strengthening in both fcc and bcc high-entropy alloys. *Acta materialia* **188**, 486-491 (2020).

7. Coury FG, Kaufman M, Clarke AJ. Solid-solution strengthening in refractory high entropy alloys. *Acta materialia* **175**, 66-81 (2019).

8. Petry W, *et al.* Phonon dispersion of the bcc phase of group-IV metals. I. bcc titanium. *Physical Review B* **43**, 10933 (1991).

9. Heiming A, *et al.* Phonon dispersion of the bcc phase of group-IV metals. II. bcc zirconium, a model case of dynamical precursors of martensitic transitions. *Physical Review B* **43**, 10948 (1991).

10. Trampenau J, *et al.* Phonon dispersion of the bcc phase of group-IV metals. III. bcc hafnium. *Physical Review B* **43**, 10963 (1991).

11. Kresse G, Furthmüller J. Efficiency of ab-initio total energy calculations for metals and semiconductors using a plane-wave basis set. *Computational Materials Science* **6**, 15-50 (1996).

12. Perdew JP, Burke K, Ernzerhof M. Generalized gradient approximation made simple. *Physical Review Letters* **77**, 3865 (1996).

13. Mihalkovič M, Widom M. Ab initio calculations of cohesive energies of Fe-based glass-forming alloys. *Physical Review B* **70**, 144107 (2004).

14. Zhang H, Yao S, Widom M. Predicted phase diagram of boron-carbon-nitrogen. *Physical Review B* **93**, 144107 (2016).

15. Gao MC, Gao P, Hawk JA, Ouyang L, Alman DE, Widom M. Computational modeling of high-entropy alloys: Structures, thermodynamics and elasticity. *Journal of Materials Research* **32**, 3627-3641 (2017).
