# Supplementary material for: Strength can be controlled by edge dislocations in refractory high-entropy alloys
Source: Nat Commun. 2021 Sep 16;12:5474. doi: 10.1038/s41467-021-25807-w (PMC8446014; doi:10.1038/s41467-021-25807-w)
Supplement: Supplementary file 3 — Description of Additional Supplementary Files [file 41467_2021_25807_MOESM3_ESM.pdf]

Deception of Additional Supplementary Files

Supplementary Data 1: The MATLAB code to predict the yield strength of BCC HEAs as a function of temperature
